# Evolution of Diagnoses, Survival, and Costs of Oncological Medical Treatment for Non-Small-Cell Lung Cancer over 20 Years in Osona, Catalonia

**DOI:** 10.3390/curroncol31040159

**Published:** 2024-04-09

**Authors:** Marta Parera Roig, David Compte Colomé, Gemma Basagaña Colomer, Emilia Gabriela Sardo, Mauricio Alejandro Tournour, Silvia Griñó Fernández, Arturo Ivan Ominetti, Emma Puigoriol Juvanteny, José Luis Molinero Polo, Daniel Badia Jobal, Nadia Espejo-Herrera

**Affiliations:** 1 Oncohematology Unit, Consorci Hospitalari de Vic (University Hospital of Vic), 08500 Vic, Spain; esardo@chv.cat (E.G.S.); matournour@chv.cat (M.A.T.); sgrino@chv.cat (S.G.F.); aiominetti@chv.cat (A.I.O.); 2 School of Medicine, University of Vic-Central University of Catalonia (UVIC-UCC), 08500 Vic, Spain; dbadia@chv.cat (D.B.J.); ncespejo@chv.cat (N.E.-H.); 3 Doctoral College, Medicine and Biomedical Sciences, University of Vic-Central University of Catalonia (UVic-UCC), 08500 Vic, Spain; 4 Mechanisms of Disease Laboratory Research Group (MoD Lab), IRIS-CC, University of Vic-Central University of Catalonia (UVic-UCC), 08500 Vic, Spain; 5 Planning and Information Systems Department, Consorci Hospitalari de Vic (University Hospital of Vic), 08500 Vic, Spain; dcompte@chv.cat; 6 Pharmacy Department, Consorci Hospitalari de Vic (University Hospital of Vic), 08500 Vic, Spain; gbasagana@chv.cat; 7 Epidemiology Department, Consorci Hospitalari de Vic (University Hospital of Vic), 08500 Vic, Spain; epuigoriol@chv.cat; 8 Multidisciplinary Inflammation Research Group (MIRG), IRIS-CC, 08500 Vic, Spain; 9 Pathology Department, Consorci Hospitalari de Vic (University Hospital of Vic), 08500 Vic, Spain; jlmolinero@chv.cat

**Keywords:** lung cancer, NSCLC: non-small-cell lung cancer, oncological treatment costs, health economics, real-world data, cancer survival

## Abstract

Non-small-cell lung cancer (NSCLC) has experienced several diagnostic and therapeutic changes over the past two decades. However, there are few studies conducted with real-world data regarding the evolution of the cost of these new drugs and the corresponding changes in the survival of these patients. We collected data on patients diagnosed with NSCLC from the tumor registry of the University Hospital of Vic from 2002 to 2021. We analyzed the epidemiological and pathological characteristics of these patients, the diverse oncological treatments administered, and the survival outcomes extending at least 18 months post-diagnosis. We also collected data on pharmacological costs, aligning them with the treatments received by each patient to determine the cost associated with individualized treatments. Our study included 905 patients diagnosed with NSCLC. We observed a dynamic shift in histopathological subtypes from squamous carcinoma in the initial years to adenocarcinoma. Regarding the treatment approach, the use of chemotherapy declined over time, replaced by immunotherapy, while molecular therapy showed relative stability. An increase in survival at 18 months after diagnosis was observed in patients with advanced stages over the most recent years of this study, along with the advent of immunotherapy. Mean treatment costs per patient ranged from EUR 1413.16 to EUR 22,029.87 and reached a peak of EUR 48,283.80 in 2017 after the advent of immunotherapy. This retrospective study, based on real-world data, documents the evolution of pathological characteristics, survival rates, and medical treatment costs for NSCLC over the last two decades. After the introduction of immunotherapy, patients in advanced stages showed an improvement in survival at 18 months, coupled with an increase in treatment costs.

## 1. Introduction

Lung cancer has been one of the most commonly diagnosed neoplasms over the last decades worldwide. In the United States, according to the latest estimates, approximately 238,340 new cases of lung cancer have been diagnosed in 2023, which account for 12.17% of the total cancer incidence [1]. This cancer has a great clinical impact due to its high mortality rate, with 127,070 deaths in 2023 worldwide (18.2% of total cancer deaths) [1,2], and it was the leading cause of cancer-related deaths worldwide (18.4% of total cancer deaths) in 2022. In Spain, it is the third most frequent cancer type in both sexes, with 31,282 new cases and 22,438 deaths in 2023 [2,3].

Specifically, the landscape of NSCLC, non-small-cell lung cancer, has undergone significant diagnostic and therapeutic transformations in the past two decades [4,5]. On the medical treatment front, numerous drugs have undergone evaluation, gaining approval from the Food and Drug Administration and securing public reimbursement from the Spanish Health Service [6]. A pivotal moment occurred in 2002, when a phase 3 study demonstrated benefits for four combined chemotherapy regimens, revealing a median survival time of approximately 8 months [7,8]. 

In 2004, pemetrexed earned approval as a novel chemotherapy agent for palliative care and subsequently was indicated for NSCLC of the non-squamous subtype [9]. In September 2013, the advent of cancer genome analyses and the identification of potential therapeutic targets produced a shift in treatment strategies [10], and the introduction of “molecular therapy” with tyrosine kinase inhibitors proved to be beneficial in terms of survival compared to conventional chemotherapy for tumors harboring an EGFR (epidermal growth factor receptor) mutation [11]. This approach was later extended to tumors with ALK (anaplastic lymphoma kinase) fusions [12] and Ros1 fusions [13]. In 2016, the introduction of immunotherapy with immune checkpoint inhibitors resulted in notable survival benefits for a subset of patients enrolled in clinical trials [14]. However, it is worth considering that results from clinical trials may not wholly reflect the actual health impact in daily clinical practice because patients included are selectively chosen and may not be fully representative of the overall cohort of lung cancer patients. Furthermore, assessing actual survival benefits in clinical trials can be challenging due to potential crossover effects among the groups of treatment [15].

On the other hand, there is a limited number of studies assessing the fluctuations in the costs and potential changes in survival rates associated with medical treatment for NSCLC, particularly considering the recent approval of more expensive drugs. Some studies have relied on simplified clinical algorithms for cost calculation, often focusing on a single line of treatment [16,17]. Other studies were conducted before the introduction of new and expensive therapeutic agents [18,19,20,21] or included the treatment cost for small cell lung cancer, which has not changed significantly over the last years [22]. In our region, only one previous study has been conducted to assess the cost of medical treatment and changes in survival among NSCLC patients based on data from tumor registries. This particular study observed an increase in treatment cost over a four-year period from 2014 to 2018 and did not show significant differences in survival rates [23].

Herein, we use real-world data from patients diagnosed with NSCLC in our region over twenty years from 2002 to 2022. Our primary objective is to delineate the evolving clinical, pathological, and therapeutic landscape of this pathology. We also describe the cost dynamics associated with pharmacological treatments and survival rates over the past two decades, comparing periods preceding and succeeding the introduction of novel therapeutic agents for NSCLC.

## 2. Materials and Methods

### 2.1. Study Design and Population

This is an observational, descriptive study that follows the STROBE recommendations [24].

We collected retrospective data from the tumor registry of the University Hospital of Vic (Consorci Hospitalari de Vic-CHV). The CHV is a health institution serving the residents of the Osona region in Catalonia, with a population of 150,000 inhabitants in 2021 [25]. The tumor registry is a repository that encompasses basic epidemiological information with other relevant variables.

From this registry, we selected patients diagnosed with NSCLC from January 2002 to December 2021. We collected epidemiological data encompassing sex, smoking habits, cancer location, age and at diagnosis, date of diagnosis, and, if applicable, date of decease. Oncological data, including histologic tumor type and initial stage (standardized to the eighth and latest edition of the TNM available until the end of 2021) [26], as well as details about the received oncological treatment, including date, treatment line, duration, and, if applicable, date of progression. Our analysis included only patients with complete information. The oncological treatments have been categorized into four blocks: conventional chemotherapy, molecular therapy, immunotherapy, and a combination of chemotherapy and immunotherapy.

### 2.2. Pharmaceutical Expenditure

We collected the invoicing prices for various drugs utilized over the study period, extracting this information from the “CAT Salut Nomenclator”, which is a comprehensive listing of all approved drugs covered by Public Health in Catalonia. The “Nomenclator” indicates the invoice price per unit (vial or tablet) for each treatment drug [27], which corresponds to the PVL price (pharmaceutical laboratory price plus 4% of taxes or IVA). While detailed retrospective data were available from 2021 back to 2012, for preceding years, we imputed the earliest available price in the “Nomenclator”. To ensure precise calculation, we created two distinct databases. The first database indicates the drugs administered in cycles, for which we computed the cost per cycle. The second database included the prices for oral drugs, and we calculated the cost per month. Subsequently, the cost databases were linked to the patient database based on the treatments administered to each patient. Costs were computed according to the price prevailing in the year of drug administration and then imputed to the year in which the patient received the diagnosis. In calculating the average annual cost, we considered only those patients who underwent medical treatment instead of the total number of patients diagnosed in a given year.

### 2.3. Statistical Analyses

We conducted a comprehensive analysis, employing various statistical methods. In the descriptive analysis, we computed frequencies and percentages for qualitative variables and measures of central tendency or dispersion for quantitative variables, including the costs of treatment.

We categorized the study population into four distinct subgroups or diagnosis periods based on the year of diagnosis: 2002 to 2006, 2007 to 2012, 2013 to 2016, and 2017 to 2022. Quantitative variables were tested for normality using the Kolmogrov–Smirnov test. To assess differences in the variables collected across diagnosis periods, we used the chi-squared test for qualitative variables and the two-way ANOVA test for quantitative variables. We conducted a survival analysis at 18 months of follow-up, utilizing the Kaplan–Meier method and Cox regression models by diagnostic period and stage. All analyses were performed using the SPSS 29.0 program. We set the statistical significance threshold at 5%.

### 2.4. Ethics

The study protocol was approved by the Ethics Committee of the University Hospital of Vic in June 2017. Informed consent was not obtained from patients, as the data utilized in the analyses were coded, and biological samples were not employed in this study. This approach ensures the privacy and confidentiality of patient information while complying with research standards. 

## 3. Results

### 3.1. Patients’ Characteristics

This study included 905 evaluable patients. Table 1 shows the overall characteristics of the population and the distribution by diagnosis period. Overall, most of the patients were men (83.8%) older than 65 years (62.1%) at the time of diagnosis. The distribution of patients according to age at diagnosis was similar across the four distinct diagnostic periods. Most of the patients were former smokers (47.5%) overall, but the category of “unknown” smoking status was more frequent in the first diagnostic period (2002–2006) compared to the latest one (2017–2021) (17% versus 4%, *p* < 0.001). The majority of patients (50.3%) were diagnosed at metastatic stage, but stage I was more frequent in the latest period, compared with the first one (16% versus 7%, *p* < 0.001). Regarding the histopathological type, most cases were classified as adenocarcinoma (42.9%) across the whole study period. Squamous carcinoma was most frequent in the first period (48.4%), whereas adenocarcinoma became the predominant type from 2012 onwards (53%), with these differences being statistically significant (*p* < 0.001).

### 3.2. Pharmacological Oncologic Treatments

Table 2 provides information on the frequency of oncological treatment across diagnostics periods. Overall, the percentage of patients treated ranged from 49.6% (2012–2016) to 63.8% (2007–2011), with no statistically significant differences observed across diagnostic periods. According to the type of treatment, the use of chemotherapy exhibited a progressive decline from 92.6% in the first period (2002–2006) to 66.9% in the latest period (2017–2021). In contrast, the use of immunotherapy increased from 2.9% in the third period (2012–2016) to 19.4% in the latest one (2017–2021) and these differences were statistically significant.

Patients in our study received a maximum of nine lines of treatment, with the majority undergoing up to three lines. A higher percentage of patients received chemotherapy as a first line of treatment during the first diagnostic period compared to the latest one (97.8% versus 73.3% *p* < 0.001). Similarly, the percentage of patients receiving chemotherapy as the second line of treatment dropped from 94.3% in the first period to 57.7% in the last one. After 2012, immunotherapy was introduced as a new treatment paradigm. In the last period, it constituted the first line of treatment for 12.9% of patients in monotherapy and 2.9% in combination with chemotherapy. Additionally, immunotherapy was applied as the second line of treatment for 30% of patients during the last period. The distribution of pharmacological treatment in the third and subsequent lines of treatment did not reach statistically significant differences due to the sample size. We observed that during the first period, from 2002 to 2006, the maximum successive line of treatment was the fifth line. Over the years, even though there are few patients, the number of treatment lines increased, mainly molecular treatment and/or immunotherapy.

### 3.3. Survival Outcomes

**Figure 1:**
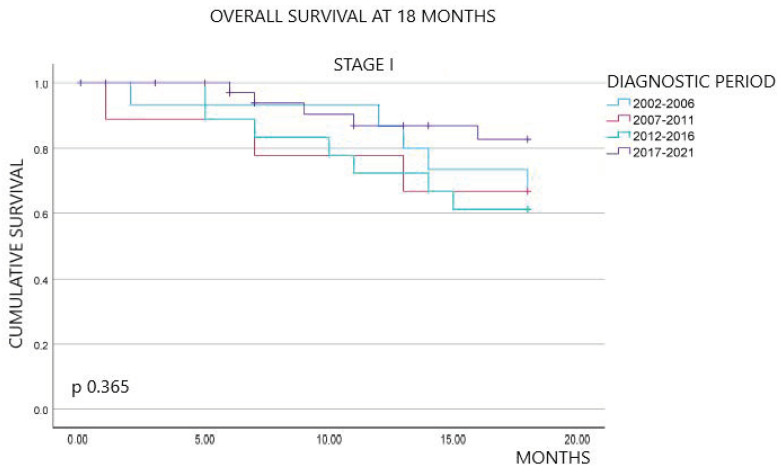

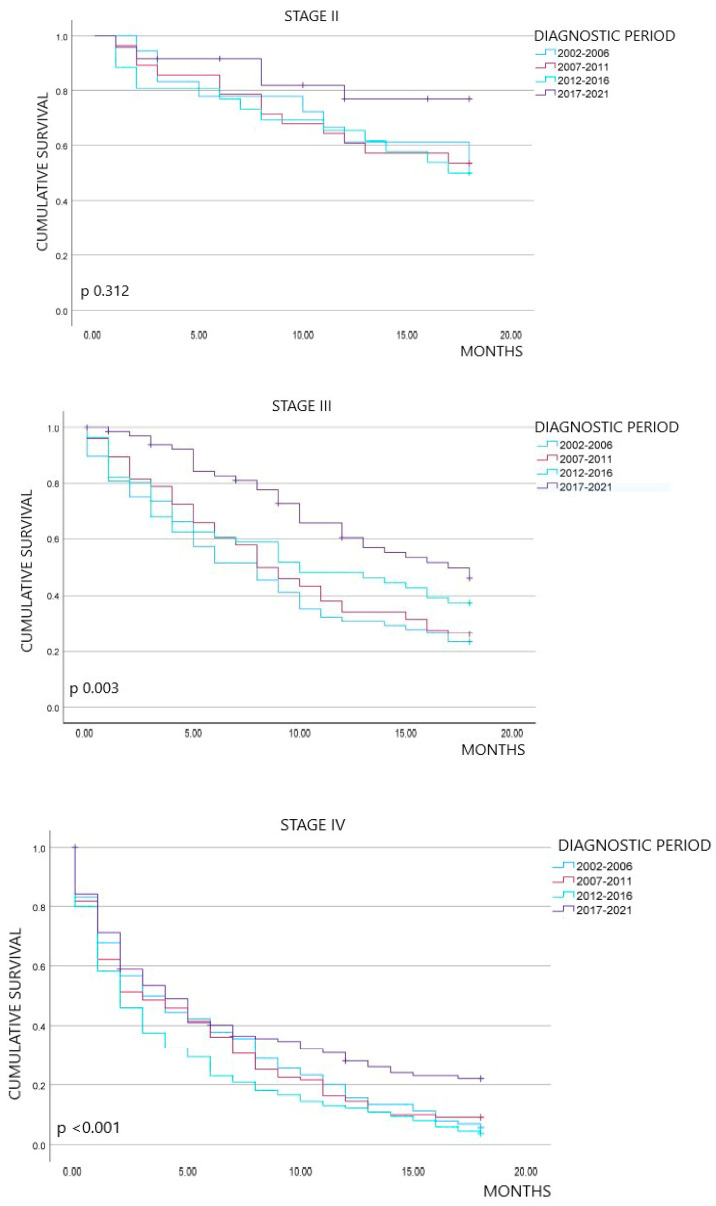
Kaplan–Meier survival curves at 18 months overall, by stage and diagnostic period.

Figure 1 shows the overall survival outcomes at 18 months of follow-up, stratified by diagnostic period and stage at diagnosis. Overall, the percentage of patients alive at 18 months of follow-up exhibited an improvement over time. Specifically, survival rates were 20.6% in the first period, 22.8% in the second, 20.8% in the third, and significantly increased to 47.4% in the last period (*p* < 0.001). By stage, we did not observe statistically significant differences between stages I and II across the four periods (*p* 0.365 and p 0.312, respectively). In contrast, survival at 18 months for stages III and IV showed an upward trend in the last period compared to the previous periods, with statistically significant differences (*p* 0.003 and *p* < 0.001, respectively).

A Cox regression analysis was conducted to identify variables associated with 18-month mortality. Notably, the year of diagnosis, age at diagnosis, smoking status, and stage were found to be associated with survival at 18 months of follow-up. In contrast, histological subtype and sex were not associated. These findings are detailed in Supplementary Table S1. 

### 3.4. Expenditure by Pharmacological Classes and Year of Diagnosis

**Figure 2:**
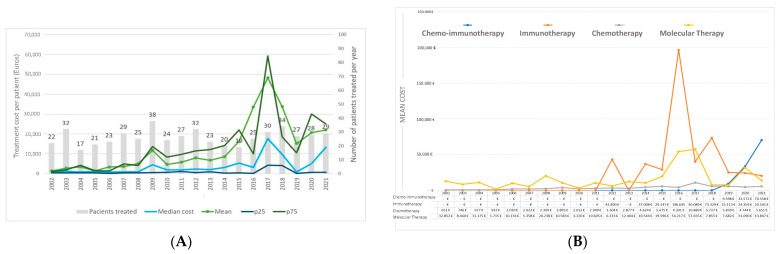
Costs of pharmacological treatment by year of diagnosis (**A**) and annual cost per patient and by type of treatment (**B**).

Figure 2 presents the pharmacological treatment costs by year of diagnosis. The average costs per patient ranged from EUR 1413.16 in 2002 to EUR 48,283.80 in 2017 (Figure 2A). Subsequently, average costs fluctuated, reaching EUR 22,029.87 per patient in 2021. The median cost followed a similar trend, with the highest observed in 2017, peaking at EUR 17,661.9 per patient. Supplementary Table S2 provides a comprehensive description of the costs of pharmacological treatment by each year of diagnosis.

Figure 2B delineates the cost of various types of treatment, offering insights into the economic dynamics associated with each modality. Chemotherapy presents a slight and progressive increase over time, ranging between EUR 651 and EUR 5651, peaking in 2009 and 2017. The costs of molecular therapy displayed oscillations ranging from EUR 12,852 to EUR 13,847. Peaks were observed in 2008, 2017, 2020, and 2022. The cost of immunotherapy as monotherapy reached its maximum in 2016 at EUR 196,640 and exhibited a subsequent decline. The cost of the combination of chemotherapy and immunotherapy experienced a progressive increase from 2019 onwards.

## 4. Discussion

This is the first study conducted in our region utilizing real-world healthcare data, detailing shifts in NSCLC diagnosis, survival rates, and pharmacological treatment costs over a long-term 20-year period. One of the relevant results is an increase in survival rates among patients in advanced stages of NSCLC in recent years, following the introduction of novel pharmacological treatments, particularly immunotherapy. The cost of medical treatment for lung cancer ranged from EUR 1143.16 in 2002 to EUR 48,283.80 in 2017, driven by the introduction of these new therapeutic agents.

Our descriptive findings of NSCLC align consistently with results from studies conducted in other countries. One of the notable results was the inversion in the frequency of squamous carcinoma versus adenocarcinoma observed over the twenty years also reported in other previous published studies [28,29]. Another notable result is the higher number of missing or “unknowns” for smoking habits between 2002 and 2006, which may be attributed to the historical context where smoking status was not considered a main determinant of lung cancer’s characteristics [30]. Regarding gender distribution, NSCLC has historically been more frequent in men, although we observed an increase in cases among women in recent diagnostic periods, with no statistically significant differences [31,32,33]. Moreover, our regression analysis did not establish a significant association between gender and survival rates. Examining the stage at diagnosis, nearly 80% of our patients were diagnosed in stage IV or III, mirroring findings available in the literature where 70% of patients are diagnosed in advanced stages [34,35]. However, the percentage of diagnoses in the early stages increased over the last 5 years of the study period. Despite the results presented in the NELSON study [36], in Catalonia, we do not have any lung cancer screening program that may be related to this increase in early diagnosis. One plausible explanation for this trend could be the incidental early diagnosis in CT scanners during the pandemic years, in patients affected by COVID-19. This interpretation is controversial since other studies reported a decrease in lung cancer diagnoses during the initial months of the pandemic [37,38].

A relevant result from this study is a statistically significant increase in survival among patients with advanced NCSLC (stages III and IV) variables associated with survival at 18 months of follow-up included the year of diagnosis and the stage at diagnosis. We performed the survival analyses stratifying by diagnostic period and stage to account for the impact of these variables on survival estimations. This analysis reveals a noteworthy increase in survival in the latest period (2017–2021), coinciding with the introduction of immunotherapy as a treatment modality. In contrast, previous studies in our region did demonstrate similar survival differences after the implementation of immunotherapy, potentially attributed to the short follow-up time after treatment administration [23]. We did not observe a statistically significant increase in survival during the initial three periods analyzed (2002–2016). Some studies reported a marginal increase in survival of 1.5 months between 2000 and 2010 [21]. These discrepancies may be attributable to the differences in sample size of the two studies. Other factors, such as the implementation of PET, may not explain the survival increase observed in our population in recent years because PET has been performed since 2008 and routinely since 2012. The improvement in surgery or radiotherapy may not influence the survival increase either because these techniques are not indicated for the treatment of patients at advanced stages of NSCLC.

This study also represents a pioneering effort to describe the dynamic changes in the costs of pharmacological treatment for NSCLC within our population over two decades. Our calculations of treatment costs were based on the PVL prices available on the website of Nomenclator. We considered these prices because they are the official costs reimbursed by the Public Health Service and standardized for all hospitals in Catalonia, regardless of the potential discounts that may be applied for each hospital. In this way, the prices we report may be more comparable with data from other hospitals in our region. Treatment costs exhibited fluctuations in keeping with evolving therapeutic approaches. From 2002 to 2007, chemotherapy and molecular therapy without specific molecular targets were the base of oncological treatment. Subsequently, between 2008 and 2012, the progressive introduction of molecular therapy for specific mutations along with pemetrexed in 2009 marked a different phase [9,38,39,40]. These changes explain the notable peak in costs during that year. After 2009, expenditures showed marginal increments. The evolving landscape of chemotherapy, with a focus on specific drugs, warrants attention in future studies, a topic not explored in the current investigation. Alongside these changes in treatment approaches, we observed an increase of EUR 4417 in the average annual cost of treatment between 2002 and 2011. These results are similar to a previous study that showed an increase in annual cost from USD 80,123 to USD 85,087 between 2000 and 2011, an increment equivalent to EUR 4592 [21]. From 2013 to 2017, molecular therapy for specific mutations became the standard. In the molecular therapy group, patient numbers have remained stable throughout the period studied, but there has been a paradigm shift towards personalized treatments guided by mutations in EGFR ALK and ROS. A similar paradigm shift was observed in previous studies [11,12,13,41,42,43,44,45,46,47,48,49,50]. 

In 2016, immunotherapy gained approval for first-line treatment and emerged as a treatment option. The landscape of available drugs and their indications in lung cancer has expanded, driven by positive outcomes in terms of survival improvement [51,52,53,54,55,56,57,58,59]. In our study, immunotherapy was prescribed for stages III and especially IV, revealing an increased survival rate at the 18-month follow-up in this group. In terms of cost, we observed a more pronounced increase during the second decade analyzed, particularly following the introduction of these immunotherapy agents. For instance, the average pharmacological treatment cost rose from EUR 8619 in 2014 to EUR 33,856 in 2018. Two previous studies conducted in Catalonia reported similar trends recently. One of them showed an increase from EUR 2923 per patient in 2014 to EUR 12,211 in 2018 [23], and the most recent one reported an increase from EUR 3545 per patient in 2010 to EUR 8371 in 2019 [60]. Our calculations may be influenced by a small number of patients receiving treatment with expensive immunotherapy agents, either alone or in combination with chemotherapy, and this is a possible explanation for discrepancies with the mentioned studies and the peak in costs observed in 2017 (see Figure 2B).

This study has limitations that we would like to acknowledge. Firstly, the relatively small sample size hampered some statistical analyses, notably survival analyses. Secondly, our study encompasses mainly populations from rural communities or small cities, potentially limiting the generalizability of our results when compared to studies conducted in populations from different environments. Thirdly, we did not account for costs derived from treatment procedures or indirect costs derived from the pathology itself for the calculation of treatment costs because this was not our primary objective, and this information was not available from a reliable source, such as “Nomenclator”. It is essential to recognize that our results may not be directly comparable to studies that include these elements in their cost calculations. Future investigations may require a more comprehensive cost analysis, incorporating additional data sources to estimate treatment costs.

Despite the limitations, this study benefits from being conducted on patients treated in a single center, employing consistent treatment protocols for all patients. The available data for a long-term study period from 2002 to 2021 provides an advantage compared with previous studies in the field. The information available enabled us to categorize our analyses into four to five-year periods, aligning with different oncologic therapeutic paradigms for NSCLC. This approach facilitated the exploration of survival changes coinciding with shifts in treatment strategies, particularly evident in the last period examined.

## 5. Conclusions

This retrospective observational study confirms that, in routine clinical practice, patients with advanced non-small-cell lung cancer have experienced therapeutic modifications, leading to a notable improvement in overall survival over recent years. However, this positive health outcome resulted in higher pharmacological costs. Immunotherapy, whether administered alone or in combination, has not only played a decisive role in the cost impact but also in the enhanced survival of these patients.

## Figures and Tables

**Table 1:** Characteristics of evaluable patients, overall and by diagnostic period.

	OVERALL N = 905	2002–2006 N = 194	2007–2011 N = 224	2012–2016 N = 240	2017–2021 N = 247	*p*-Value
**SEX**						0.221 *
**Men**	758 (83.8%)	165 (85.1%)	195 (87.1%)	200 (83.3%)	198 (80.2%)	
**Women**	147 (16.2%)	29 (14.9%)	29 (12.9%)	40 (16.7%)	49 (19.8%)	
**SMOKING HABIT**						<0.001 *
**Former smoker**	430 (47.5%)	89 (45.9%)	103 (46.0%)	119 (49.6%)	119 (48.2%)	
**Smoker**	302 (33.4%)	50 (25.8%)	82 (36.6%)	86 (35.8%)	84 (34.0%)	
**Non-smoker**	109 (12.0%)	22 (11.3%)	29 (12.9%)	24 (10.00%)	34 (13.8%)	
**Unknown**	64 (7.1%)	33 (17.0%)	10 (4.5%)	11 (4.6%)	10 (4.0%)	
**AGE AT DIAGNOSIS**						0.402 **
**Mean (SD)**	67.8 ± 10.7	66.6 ± 11.6	68.1 ± 11.2	68.2 ± 10.8	68.0 ± 9.2	
**Up to 65 years**	343 (37.9%)	78 (40.2%)	81 (63.2%)	90 (37.5%)	94 (38.1%)	0.729 *
**65 to 74 years**	296 (32.7%)	60 (30.9%)	72 (32.1%)	70 (29.2%)	94 (38.1%)	
**75 to 79 years**	137 (15.1%)	33 (17.0%)	34 (15.2%)	42 (17.5%)	28 (11.3%)	
**More than 80 years**	129 (14.3%)	23 (11.9%)	37 (16.5%)	38 (15.8%)	31 (12.6%)	
**TUMOR TYPE**						<0.001 *
**Squamous**	344 (38.0%)	94 (48.5%)	86 (38.4%)	79 (32.9%)	85 (34.4%)	
**Adenocarcinoma**	388 (42.9%)	48 (24.7%)	83 (37.1%)	127 (52.9%)	130 (52.6%)	
**Non-small cell (NOS)**	93 (10.3%)	27 (13.9%)	29 (12.9%)	12 (5.0%)	25 (10.1%)	
**Other**	80 (8.8%)	25 (12.9%)	26 (11.6%)	22 (9.2%)	7 (2.8%)	
**STAGE**						<0.001 *
**Stage I**	82 (9.1%)	15 (7.7%)	9 (4.0%)	18 (7.5%)	40 (16.2%)	
**Stage II**	96 (10.6%)	18 (9.3%)	28 (12.5%)	26 (10.8%)	24 (9.7%)	
**Stage III**	267 (29.5%)	68 (35.1%)	76 (33.9%)	56 (23.3%)	67 (27.1%)	
**Stage IV**	455 (50.3%)	90 (46.4%)	111 (49.6%)	139 (57.9%)	116 (46.6%)	
**Not Defined**	5 (0.6%)	3 (1.5%)	0 (0.0%)	1 (0.4%)	1 (0.4%)	

SD: standard deviation. NOS: not otherwise specified. * *p*-values for linear-by-linear association test (Chi^2^) for differences by diagnostic period. ** *p*-value for two-way ANOVA test.

**Table 2:** Summary of oncological treatments administered overall and by diagnostic period.

Diagnostic Period Total Patients with NSCLC Diagnosis	2002–2006 194	2007–2011 224	2012–2016 240	2017–2021 247	*p*-Value *
**Total patients medically treated**	115 (59.3%)	143 (63.8%)	119 (49.6%)	148 (59.9%)	0.418
**Total number of times that any medical treatment was administered**	**N = 175** **(100%)**	**N = 259** **(100%)**	**N = 208** **(100%)**	**N = 278** **(100%)**	
**Type of treatment administered overall**					
Chemotherapy	162 (92.6%)	223 (86.1%)	183 (88.0%)	186 (66.9%)	**<0.001**
Molecular therapy	13 (7.4%)	36 (13.9%)	19 (9.1%)	33 (11.9%)	0.483
Immunotherapy	0	0	6 (2.9%)	54 (19.4%)	**<0.001**
Chemo + immunotherapy	0	0	0	5 (1.8%)	-
**Type of treatment administered by lines**					
**First line**	**94 (48.4%)**	**112 (50.0%)**	**79 (32.9%)**	**101 (40.7%)**	**0.008**
Chemotherapy	92 (97.8%)	101 (90.2%)	75 (94.9%)	74 (73.3%)	**<0.001**
Molecular therapy	2 (2.2%)	11 (9.8%)	4 (5.1%)	11 (10.9%)	
Immunotherapy	0	0	0	13 (12.9%)	
Chemo + immunotherapy	0	0	0	3 (2.9%)	
**Second line**	**53 (27.3%)**	**73 (32.5%)**	**72 (30.0%)**	**97 (39.1%)**	**0.017**
Chemotherapy	50 (94.3%)	63 (86.3%)	66 (91.7%)	56 (57.7%)	**<0.001**
Molecular therapy	3 (5.7%)	10 (13.7%)	6 (8.3%)	10 (10.3%)	
Immunotherapy	0	0	0	29 (30.0%)	
Chemo + immunotherapy	0	0	0	2 (2.0%)	
**Third line**	**16 (8.2%)**	**39 (17.4%)**	**29 (12.1%)**	**40 (16.1%)**	0.096
Chemotherapy	12 (75.0%)	30 (76.9%)	22 (75.9%)	31 (77.5%)	0.072
Molecular therapy	4 (25.0%)	9 (23.1%)	4 (13.8%)	3 (7.5%)	
Immunotherapy	0	0	3 (10.3%)	6 (15.0%)	
**Fourth line**	**9 (4.6%)**	**17 (7.5%)**	**16 (6.6%)**	**21 (8.0%)**	0.177
Chemotherapy	7 (77.8%)	16 (94.1%)	14 (87.4%)	14 (66.7%)	0.053
Molecular therapy	2 (22.2%)	1 (5.9%)	1 (6.3%)	3 (14.3%)	
Immunotherapy	0	0	1 (6.3%)	4 (19.0%)	
**Fifth line**	**3 (1.5%)**	**10 (4.4%)**	**5 (2.1%)**	**15 (6.0%)**	0.038
Chemotherapy	1 (33.3%)	8 (80.0%)	5 (100.0%)	9 (60.0%)	0.519
Molecular therapy	2 (66.6%)	2 (20.0%)	0	4 (26.7%)	
Immunotherapy	0	0	0	2 (13.3%)	
**Sixth line**	0	**5 (2.2%)**	**3 (1.2%)**	**3 (1.2%)**	0.503
Chemotherapy	0	3 (60.0%)	0	2 (60.0%)	0.884
Molecular therapy	0	2 (40.0%)	2 (60%)	1 (40.0%)	
Immunotherapy	0	0	1 (40%)	0	
**Seventh line**	0	**3 (1.3%)**	**2 (0.8%)**	**1 (0.4%)**	0.876
Chemotherapy	0	2 (60.0%)	1 (50.0%)	0	0.317
Molecular therapy	0	1 (40.0%)	1 (50.0%)	1 (100%)	
**Eighth line**	0	0	**1 (0.4%)**	0	-
Immunotherapy	0	0	1 (100.0%)	0	-
**Ninth line**	0	0	**1 (0.4%)**	0	-
Molecular therapy	0	0	1 (100.0%)	0	-

* *p*-values for linear-by-linear association test (Chi^2^).

## Data Availability

The study data are available from the corresponding author upon request.

## Supplementary Materials

The following supporting information can be downloaded at https://www.mdpi.com/article/10.3390/curroncol31040159/s1, Table S1: Factors associated with mortality at 18 months and their hazard ratios, Table S2: Description of costs of medical treatment per year of diagnosis.
