# Supplementary material for: Evolution of Diagnoses, Survival, and Costs of Oncological Medical Treatment for Non-Small-Cell Lung Cancer over 20 Years in Osona, Catalonia
Source: Curr Oncol. 2024 Apr 9;31(4):2145–57. doi: 10.3390/curroncol31040159 (PMC11049066; doi:10.3390/curroncol31040159)
Supplement: Supplementary file 1 [file curroncol-31-00159-s001.zip › curroncol-2935384-supplementary.pdf]

SUPPLEMENTARY MATERIALS:

Supplementary Table S1. Factors associated with mortality at 18 months and their Hazard Ratios.

Variable	Categories	Hazard Ratio (CI 95%)
Diagnosis year	2002-2006	1
	2007-2011	0,952 (0,761 – 1,191)
	2012-2016	1,032 (0,827 – 1,287)
	2017-2021	0,590 (0,463 – 0,752)
Age at diagnosis	< 65 years	1
	65 – 74 years	1,239 (1,021 – 1,504)
	75 – 79 years	1,367 (1,080 – 1,731)
	>80 years	2,068 (1,625 – 2,632)
Smoking habit	Current smoker	1
	Former smoker	0,918 (0,765 – 1,1052)
	Non-smoker	0,571 (0,430 – 0,757)
	Unknown	1,143 (0,829 – 1,576)
Stage at diagnosis	I	1
	II	1,620 (0,945 – 2,777)
	III	3,333 (2,095 – 5,302)
	IV	7,841 (4,985 – 12,332)

Supplementary Table S2. Total, mean, and median costs per year of diagnosis.

Diagnosis year	Medically treated patients	Total Cost per year	Mean cost (SD)	Median cost (p25-p75)
2002	22	31.089,50 €	1.413,16 € (3.106,7)	651,0€ (393-1.182)
2003	32	90.273,40 €	2.821,04 € (4.263,9)	1.222,0€ (438-1.945)
2004	17	59.746,33 €	3.514,49 € (5.361,9)	868,0€ (434-4.311)
2005	21	30.478,07 €	1.451,34 € (1.814,2)	1142,0€ (420-1.577)
2006	23	79.141,23 €	3.440,92 € (8.888,6)	868,0€ (230-1.406)
2007	29	104.414,10 €	3.600,49 € (5.961,2)	920,0€ (434-4.926)
2008	25	125.918,20 €	5.036,73 € (10.879,1)	1.071,0€ (434-4.110)
2009	38	448.136,63 €	11.793,07 € (21.355,1)	4539,3€ (562-13.691)
2010	24	112.678,27 €	4.694,93 € (5.163,2)	2.019,5€ (749-8.333)
2011	27	157.433,13 €	5.830,86 € (7.506.7)	2.007,0€ (1.132-9.666)
2012	32	255.950,40 €	7.998,45 € (14.372,2)	2.364,5€ (651-11.463)
2013	23	156.068,33 €	6.785,58 € (8.452,9)	2.095,0€ (981-12.198)
2014	20	172.394,60 €	8.619,73 € (12.162.1)	3.142.5€ (336-14.147)
2015	19	308.693,53 €	16.247,03 € (24.917,5)	5.338.0€ (508-21.944)
2016	25	841.849,43 €	33.673,98 € (117.072,4)	3.182,0€ (244-9.997)
2017	30	1.448.513,87 €	48.283,80 € (45.990,5)	17.661,9€ (4.223-59.264)

2018	34	1.151.108,87 €	33.856,14 € (28.968,3)	9.672,5€ (4.144-18.573)
2019	27	410.592,50 €	15.207,13 € (17.612,5)	1.062.3€ (244-10.578)
2020	28	578.502,83 €	20.660,82 € (22.205,3)	5.117,5€ (706-30.097)
2021	29	638.866,17 €	22.029,87 € (26.123,6)	13.300,0€ (728-25.120)
